# Association of preoperative retinal microcirculation and perioperative outcomes in patients undergoing congenital cardiac surgery

**DOI:** 10.1186/s13023-023-02969-y

**Published:** 2023-12-08

**Authors:** Cong Li, Zhuoting Zhu, Haiyun Yuan, Yijun Hu, Yunlian Xue, Pingting Zhong, Manqing Huang, Yun Ren, Yu Kuang, Xiaomin Zeng, Honghua Yu, Xiaohong Yang

**Affiliations:** 1 Guangdong Eye Institute, Department of Ophthalmology, Guangdong Provincial People’s Hospital (Guangdong Academy of Medical Sciences), Southern Medical University, Guangzhou, China; 2 School of Medicine, South China University of Technology, Guangzhou, China; 3 Department of Cardiovascular Surgery, Guangdong Provincial Key Laboratory of South China Structural Heart Disease, Guangdong Cardiovascular Institute, Guangdong Provincial People’s Hospital, Guangdong Academy of Medical Sciences, Guangzhou, China; 4 Statistics Section, Information Management Department, Guangdong Provincial People’s Hospital, Guangdong Academy of Medical Sciences, Guangzhou, China; 5 Zhongshan Ophthalmic Center, State Key Laboratory of Ophthalmology, Sun Yat-Sen University, Guangzhou, China; 6 Medical College, Shantou University, Shantou, China; 7 The Second School of Clinical Medicine, Southern Medical University, Guangzhou, China; 8 Guangdong Provincial Key Laboratory of Artificial Intelligence in Medical Image Analysis and Application, Guangzhou, China

**Keywords:** Congenital Heart Disease, Cardiac Surgery, Perioperative outcomes, Retinal vessel density, Optical coherence tomography angiography

## Abstract

**Background:**

Microcirculatory dysfunction is associated with increased morbidity and mortality in cardiac surgery patients. This study aimed to investigate the association between preoperative retinal microcirculation evaluated using optical coherence tomography angiography (OCTA) and perioperative outcomes in patients with congenital heart disease (CHD).

**Methods:**

This prospective, observational study was performed from May 2017 to January 2021. OCTA was used to automatically quantify the vessel density (VD) of the superficial capillary plexus, deep capillary plexus (DCP), and radial peripapillary capillary (RPC) preoperatively. The primary outcome was excessive postoperative bleeding, defined as bleeding volume > 75th percentile for 24-hour postoperative chest tube output. The secondary outcome was composite adverse outcomes, including one or more operative mortalities, early postoperative complications, and prolonged length of stay. The association between retinal VD and outcomes was assessed using Poisson regression.

**Results:**

In total, 173 CHD patients who underwent cardiac surgery were included (mean age, 26 years). Among them, 43 (24.9%) and 46 (26.6%) developed excessive postoperative bleeding and composite adverse outcomes, respectively. A lower VD of DCP (odds ratio [OR], 1.24; 95% confidence interval [CI], 1.08–1.43; *P* = 0.003) was independently associated with excessive postoperative bleeding, and a lower VD of RPC (OR, 1.97; 95% CI, 1.08–3.57; *P* = 0.027), and DCP (OR, 2.17; 95% CI, 1.08–4.37; *P* = 0.029) were independently associated with the postoperative composite adverse outcomes.

**Conclusion:**

Preoperative retinal hypoperfusion was independently associated with an increased risk of perioperative adverse outcomes in patients with CHD, suggesting that retinal microcirculation evaluation could provide valuable information about the outcomes of cardiac surgery, thereby aiding physicians in tailoring individualized treatment.

**Supplementary Information:**

The online version contains supplementary material available at 10.1186/s13023-023-02969-y.

## Background

Congenital heart disease (CHD) is the most common congenital anomaly and accounts for nearly one-third of all congenital defects, representing a global public health problem and placing a heavy burden on society and families [1, 2]. Although postoperative mortality has substantially decreased with the dramatic improvement of diagnostic and treatment capabilities for CHD [3], perioperative management and outcome prediction remain challenging. The comprehensive preoperative assessment aimed at identifying patients in need of critical perioperative management and at high risk of adverse outcomes could inform individualized treatment strategies, significantly reducing morbidity and costs. Impaired microcirculatory perfusion is reportedly independently associated with adverse outcomes, such as bleeding and complications, in patients with valvular or coronary diseases undergoing cardiac surgery with cardiopulmonary bypass (CPB) [4–6], implying that preoperative microcirculation evaluation might provide additional information when predicting surgical outcome [7, 8].

Retinal circulation is a part of human circulation, providing a unique and easily accessible window to evaluate systemic microvascular pathology in patients undergoing cardiac surgery [9, 10]. Optical coherence tomography angiography (OCTA) is a noninvasive and rapid imaging technique used to obtain high-resolution, three-dimensional images of the retinal microvasculature without intravenous dye injection and can automatically quantify retinal microcirculatory perfusion at different layers [11]. In recent years, OCTA has been widely used to monitor microvascular function in systemic diseases such as coronary artery disease [12], neurodegenerative disorders [13], and chronic kidney disease [14]. We previously identified an impairment of the retinal microcirculation in CHD patients using OCTA examination, which subsequently improved after congenital cardiac surgery [15, 16]. However, the relationship between retinal microcirculation and the clinical outcomes of congenital cardiac surgery has not been fully explored.

Therefore, we aimed to investigate the association between preoperative retinal microcirculation and perioperative outcomes in CHD patients undergoing cardiac surgery with CPB. We hypothesized that preoperative retinal hypoperfusion was independently related to adverse perioperative outcomes in CHD patients.

## Methods

### Study design and patient population

This prospective observational cohort study was approved by the Research Ethics Committee of Guangdong Provincial People’s Hospital (GDPH) [No.GDREC2018148H(R1)], and all study procedures were performed in accordance with the principles of the Declaration of Helsinki. Written informed consent was obtained from all study participants (or their parents or legal guardians, in the case of children under 16 years of age) prior to the study. This study followed the Strengthening the Reporting of Observational Studies in Epidemiology (STROBE) reporting guideline.

The study was performed from May 2017 to January 2021 in the Department of Cardiovascular Surgery at GDPH, a tertiary hospital and key cardiovascular institute in South China. The recruited participants were patients with CHD aged ≥ 5 years who were scheduled for congenital cardiac surgery with CPB in GDPH. All patients underwent OCTA before cardiac surgery.

The exclusion criteria were as follows: (1) pre-existing coagulopathy, liver dysfunction, or kidney disease; (2) no CPB performed during surgery; (3) uncooperative with ophthalmic examinations; (4) media opacities preventing high-quality imaging; (5) a history of glaucoma, retinal diseases, or intraocular surgery; and (6) comorbidities, such as hypertension, diabetes, coronary heart disease, and renal disease.

### Optical coherence tomographic angiography

OCTA is a non-invasive imaging modality that provides three-dimensional structural and deep-resolved angiographic information of the retina. All patients underwent a 6 × 6 mm^2^ high-definition (HD) macular scan and a 4.5 × 4.5 mm^2^ HD optic disc scan by the same trained ophthalmic technician. The AngioVue OCTA system (Version 2017.1.0.151; RTVue-XR Avanti; Optovue, Fremont, CA, USA) used a light source with a peak wavelength of 840 nm, bandwidth of 50 nm, scanning speed of 70 kHz, and axial tissue resolution of 5 μm. The three-dimensional Projection Artifact Removal (3D PAR) was equipped to reduce projection artifacts in the deeper layers while maintaining their authentic layout.

A series of quantitative OCTA metrics was automatically measured using the built-in split-spectrum amplitude-decorrelation angiography (SSADA) software algorithm, including the vessel density (VD) of the superficial capillary plexus (SCP), SCP-parafovea, SCP-perifovea, deep capillary plexus (DCP), DCP-parafovea, DCP-perifovea, radial peripapillary capillary (RPC), and peripapillary RPC (Supplementary Figure S1). The capillary density of the RPC and peripapillary RPC was measured with a built-in feature called large-vessel masking, which removes larger vessels (diameter ≥ 33 μm) for the 4.5 × 4.5 mm^2^ HD optic disc scan. The SCP was delineated by the internal limiting membrane (ILM) to 10 μm above the inner plexiform layer (IPL) and the DCP was delineated by 10 μm above the IPL to 10 μm below the outer plexiform layer (OPL). The parafovea referred to the area between the 1–3 mm concentric ring center of the fovea, while the perifovea referred to the 3–6 mm concentric ring center of the fovea. The RPC was delineated by the layer between the outer limit of the retinal nerve fiber layer (RNFL) and ILM, and the peripapillary region was delineated by 2- and 4-mm-diameter elliptical contour lines around the optic disc boundary. VD was defined as the percentage of the measured area occupied by the flowing blood vessels. The high repeatability and reproducibility of this OCTA instrument for these measurements have been previously demonstrated [17, 18].

Both eyes of the patients underwent OCTA, but only the data from one eye were randomly selected for analysis. All of the OCTA images were then further quality controlled with the following exclusion criteria: (1) quality index (QI) < 6, (2) residual motion artifacts, (3) segmentation error, and (4) local weak signal caused by artifacts such as floaters.

### Surgical procedures

Preoperative anesthesia management, surgical procedures (including sternotomy, CPB establishment, correction of congenital cardiac defects, chest closure, and skin suture), and postoperative intensive care unit (ICU) management were performed following the standard practice of GDPH. First, heparin was used to maintain an activated clotting time of ≥ 480 s before CPB initiation. Second, the diluted hematocrit was generally maintained at 25–30% during CPB, and CPB was generally conducted under mild hypothermia (30–34 °C) according to the surgical requirements. Standard ultrafiltration was performed routinely, and intermittent antegrade modified St. Thomas cardioplegia was used to protect the myocardium. All surgical procedures were performed by the same team, and patients with CHD were rewarmed to a bladder temperature > 35 °C before separation from CPB. Finally, heparin was neutralized by protamine administration after separation. All patients were transferred to the ICU and mechanically ventilated postoperatively.

### Data collection

Data collected included demographics, preoperative characteristics, preopeative laboratory parameters (haemoglobin, haematocrit, activated partial thromboplastin time, prothrombin time, thrombin time, international normalized ratio and fibrinogen), details of the operation, and early postoperative outcomes in all CHD patients. Cyanosis was defined as a preoperative resting oxygen saturation ≤ 90% as measured by pulse oximetry [19]. Surgical complexity was stratified using the Society of Thoracic Surgeons-European Association for Cardio-Thoracic Surgery (STAT) Mortality Categories [20]. The surgical procedures were assigned to one of the five categories based on a similar risk of in-hospital mortality, with category 1 having the lowest risk of death, and category 5 having the highest risk. If multiple surgeries were performed, only the first surgery was included. For multicomponent operations, the primary procedure with the highest STAT score was identified. We collapsed STAT categories 1–3 as lower-risk cases because of the small sample size of STAT category 3 [21].

### Outcome measures

The primary perioperative outcome was excessive postoperative bleeding, which was defined as bleeding volume > 75th percentile for 24-hour postoperative chest tube output (CTO) [22]. The secondary outcome was composite adverse outcomes including in-hospital mortality, early postoperative complications, and prolonged length of stay. The in-hospital mortality was defined as all deaths occurring during hospitalization or after discharge but before the end of the 30th postoperative day; the early postoperative complications were determined using the Society of Thoracic Surgeons Congenital Heart Surgery Database definitions; and the prolonged length of stay was defined as the postoperative length of stay > 14 days [23]. The Society of Thoracic Surgeons Congenital Heart Surgery database collected perioperative information on all patients at participating institutions undergoing CHD operations. Approximately three-quarters of all centers performing congenital heart surgery submit data to this database. The early postoperative complications (which occurred within 30 days of surgery) were defined by the database in a standard fashion based on a review of the available literature on the durable impact of these complications on long-term outcomes, including unplanned readmission, respiratory insufficiency, pneumonia, chylothorax or pneumothorax, and arrhythmia, etc. A composite adverse outcome was chosen because the perioperative mortality in our study was relatively low. A higher event rate of the composite outcome was expected to improve the statistical power of the study.

### Statistical analysis

The variables were tested for normality using the Shapiro-Wilk test and summarized as the means ± standard deviations (SDs) or medians (interquartile ranges, [IQR]) for continuous variables and numbers (percentages) for categorical variables. For comparisons of continuous data, an independent Student’s t-test or Mann-Whitney test was used, and for comparisons of categorical data, a χ^2^ test or Fisher’s exact test was used. As there was no definite cut-off value for retinal vessel density (RVD), receiver operating characteristic (ROC) analyses were performed to categorize RVD according to the Youden index (Supplementary Figures S2 and S3) [24]. Poisson regression was used to assess the association between RVD and perioperative outcomes. Three models were established using Poisson regression: Model 1 was the base model; Model 2 was adjusted for demographic factors, including age, sex, and BMI; and Model 3 included Model 2 plus the factors that were considered clinically relevant or significantly associated with outcomes in the univariate models. Odds ratios (ORs) and 95% confidence intervals (CIs) were also calculated. Statistical analyses were performed using SPSS (version 25.0, SPSS. Inc., Chicago, IL, USA) and MedCalc (version 15.2.2, Ostend, Belgium). Statistical significance was determined as *P* < 0.05 based on two-sided tests.

## Results

### Patient characteristics

**Fig. 1:**
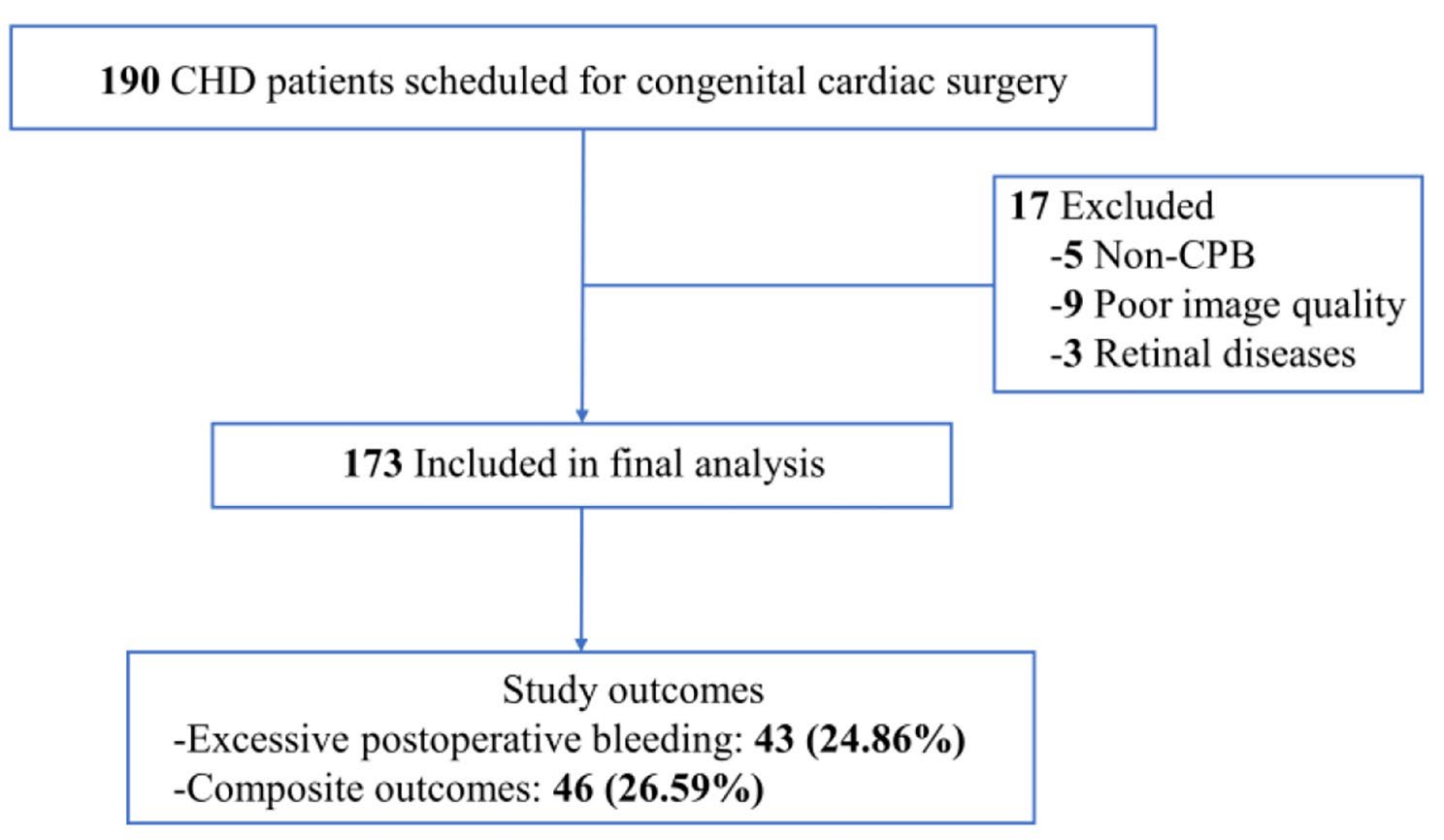
Flow chart Abbreviations: CHD, congenital heart disease; CPB, cardiopulmonary bypass

In total, 190 CHD patients scheduled for congenital cardiac surgery were recruited for this study. We excluded 17 patients (detailed reasons for excluding them from the analysis are provided in Fig. 1), leaving 173 patients with CHD in the final analysis. The baseline patient characteristics are summarized in Table 1. The mean (SD) age of the participants was 26 (13) years (range, 5–64 years), and 42.2% were males. Among them, 43 (24.9%) and 46 (26.6%) developed excessive postoperative bleeding and composite adverse outcomes, respectively.

**Table 1:** Baseline characteristics of patients (n = 173)

	Value
Age, years	26 ± 13
Gender, n (%)	
Male	73(42.2)
Female	100(57.8)
Body mass index, kg/m^2^	18.9 ± 3.7
Previous sternotomy, %	23(13.3)
Cyanosis, n (%)	44(25.4)
Preoperative SaO_2_, (%)	98(89–100)
Main diagnoses, n (%)	
Atrial septal defect	35(20.2)
Ventricular septal defect	12(6.9)
Atrioventricular septal defect	10(5.8)
Ebstein anomaly	30(17.3)
Tetralogy of Fallot	25(14.5)
Single ventricle	9(5.2)
Double-outlet right ventricle	6(3.5)
Stenosis of right ventricular outflow tract	6(3.5)
Pulmonary atresia	5(2.9)
Anomalous pulmonary venous connection	5(2.9)
Other	30(17.3)
Preoperative laboratory parameters	
Hb, g/L	142(129–163)
HCT, L/L	0.4(0.4–0.5)
APTT, s	41.1(37.6–44.2)
PT, s	14.2(13.7–15.2)
TT, s	16.5(15.8–17.1)
INR	1.1(1.0-1.2)
Fibrinogen, g/L	2.5(2.2-3.0)
Operation complexity by STAT mortality category, n (%)	
1	40(23.1)
2	90(52.0)
3	4(2.3)
4	39(22.6)
Operation time, minutes	232(180–292)
CPB time, minutes	120(92–169)
ACC time, minutes	68(43–95)
24-hour CTO, mL	293(200–424)
Excessive postoperative bleeding, n (%)	43(24.9)
ICU stay, days	2(1–4)
Mechanical ventilation time, hours	7(4–20)
PLOS, days	7(6–12)
Prolonged PLOS, n (%)	26(15.0)
Composite adverse outcomes, n (%)	46(26.6)
Postoperative complication, n (%)	
Pneumonia	9(5.2)
Respiratory insufficiency	7(4.1)
Chylothorax/pneumothorax	4(2.3)
Arrhythmia	3(1.7)
Wound dehiscence	2(1.2)
Delayed sternum closure	2(1.2)
Unplanned readmission	2(1.2)
Cardiac arrest	1(5.8)
Other	6(3.5)
Operative mortality	3(1.7)

Continuous variables are expressed as mean ± standard deviation or median (interquartile range), and categorical variables are expressed as percentages of total

Abbreviations: SaO_2_, oxygen saturation; Hb, haemoglobin; HCT, haematocrit; APTT, activated partial thromboplastin time; PT, prothrombin time; TT, thrombin time; INR, international normalized ratio; STAT, Society of Thoracic Surgeons-European Association for Cardio-Thoracic Surgery; CPB, cardiopulmonary bypass; ACC, aortic cross-clamp; CTO, chest tube output; ICU, intensive care unit; PLOS, postoperative length of stay

### Association between RVD and excessive postoperative bleeding

**Fig. 2:**
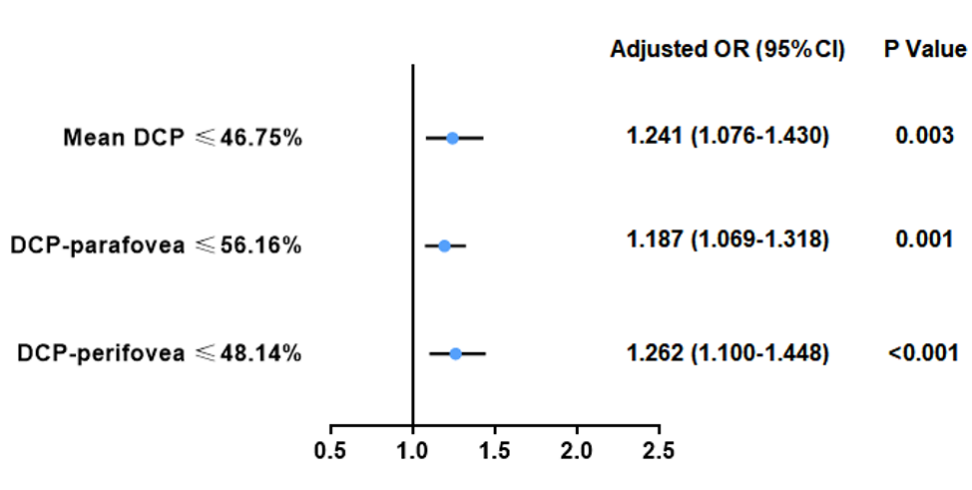
Forest plot for the presence of excessive postoperative bleeding. Adjusted variables: age, sex, body mass index, cyanosis, STAT mortality category, cardiopulmonary bypass time, haematocrit and international normalized ratio Abbreviations: DCP, deep capillary plexus; OR, odds ratio; CI, confidence intervals; STAT, Society of Thoracic Surgeons-European Association for Cardio-Thoracic Surgery Congenital Heart Surgery

Supplementary Table S1 shows the clinical characteristics of patients with CHD with or without excessive postoperative bleeding. The median (IQR) of 24-hour CTO was 293 (200–424) in all patients, 600 (460–875) in the bleeder group, and 250 (155–323) in the non-bleeder group, respectively. Compared with the non-bleeder group, the bleeder group had a higher proportion of cyanosis, higher haemoglobins, haematocrits and international normalized ratio, and longer operation, CPB and aortic cross-clamp times (all *P* < 0.05). In addition, the bleeder group had a longer ICU stay, longer mechanical ventilation time, and longer postoperative length of stay (all *P* < 0.01). In the comparison of RVD between the non-bleeder and bleeder groups, the VD of DCP, DCP-parafovea, and DCP-perifovea were significantly decreased in the bleeder group (all *P* < 0.05). The VD of RPC and SCP were comparable between the non-bleeder and bleeder groups (all *P* > 0.05) (Table 2). The association between DCP VD and excessive postoperative bleeding is presented in Table 3. After adjusting for confounding factors including age, sex, BMI, cyanosis, STAT mortality category, CPB time, haematocrit and international normalized ratio, lower VD of the DCP (OR, 1.24; 95% CI, 1.08–1.43; *P* = 0.003), DCP-parafovea (OR, 1.19; 95% CI, 1.07–1.32; *P* = 0.001), and DCP-perifovea (OR, 1.26; 95% CI, 1.10–1.45; *P* < 0.001) were still significantly associated with the presence of excessive postoperative bleeding (Fig. 2).

**Table 2:** Comparison of preoperative RVD in patients with and without excessive postoperative bleeding

	Bleeder (n = 43)	Non-bleeder (n = 130)	*P* value
**RPC density, %**			
Mean	54.61 ± 3.35	55.09 ± 3.08	0.381
Peripapillary	56.02 ± 4.06	56.30 ± 3.71	0.675
**RPC capillary density, %**			
Mean	46.88 ± 4.30	47.72 ± 3.53	0.199
Peripapillary	48.18 ± 5.45	48.73 ± 4.35	0.510
**Macular vessel density, %**			
Mean SCP	50.52 ± 3.21	50.49 ± 2.52	0.950
SCP-parafovea	53.15 ± 3.28	53.09 ± 2.98	0.924
SCP-perifovea	51.21 ± 3.40	51.19 ± 2.76	0.973
Mean DCP	50.76 ± 6.36	53.24 ± 5.17	0.011*
DCP-parafovea	54.85 ± 5.58	56.89 ± 4.42	0.015*
DCP-perifovea	51.88 ± 6.89	54.69 ± 5.49	0.018*

Abbreviations: RVD, retinal vessel density; RPC, radial peripapillary capillary; SCP, superficial capillary plexus; DCP, deep capillary plexus

*Significant statistical difference, *P* < 0.05

**Table 3:** Association between binary retinal vessel density and the presence of excessive postoperative bleeding

	Model 1		Model 2		Model 3	
	OR (95% CI)	P value	OR (95% CI)	P value	OR (95% CI)	P value
**Mean DCP, %**						
High (> 46.75)	Reference		Reference		Reference	
Low (≤ 46.75)	1.25 (1.09–1.43)	0.001*	1.26 (1.09–1.45)	0.002*	1.24 (1.08–1.43)	0.003*
**DCP-parafovea, %**						
High (> 56.16)	Reference		Reference		Reference	
Low (≤ 56.16)	1.20 (1.08–1.32)	< 0.001*	1.20 (1.08–1.32)	0.001*	1.19 (1.07–1.32)	0.001*
**DCP-perifovea, %**						
High (> 48.14)	Reference		Reference		Reference	
Low (≤ 48.14)	1.27 (1.11–1.45)	< 0.001*	1.28 (1.11–1.47)	< 0.001*	1.26 (1.10–1.45)	< 0.001*

Model 1: univariate; Model 2: adjusted for age, sex and body mass index; Model 3: adjusted each factor for model 2 plus cyanosis, STAT mortality category, cardiopulmonary bypass time, haematocrit and international normalized ratio

Retinal vessel density is divided into two categories according to cut-off points at the point of maximal sum of sensitivity and specificity using the receiver operating characteristic (ROC) curve

Abbreviations: DCP, deep capillary plexus; OR, odds ratio; CI, confidence intervals; STAT, Society of Thoracic Surgeons-European Association for Cardio-Thoracic Surgery

*Significant statistical difference, *P* < 0.05

### Association between RVD and the composite adverse outcomes

**Fig. 3:**
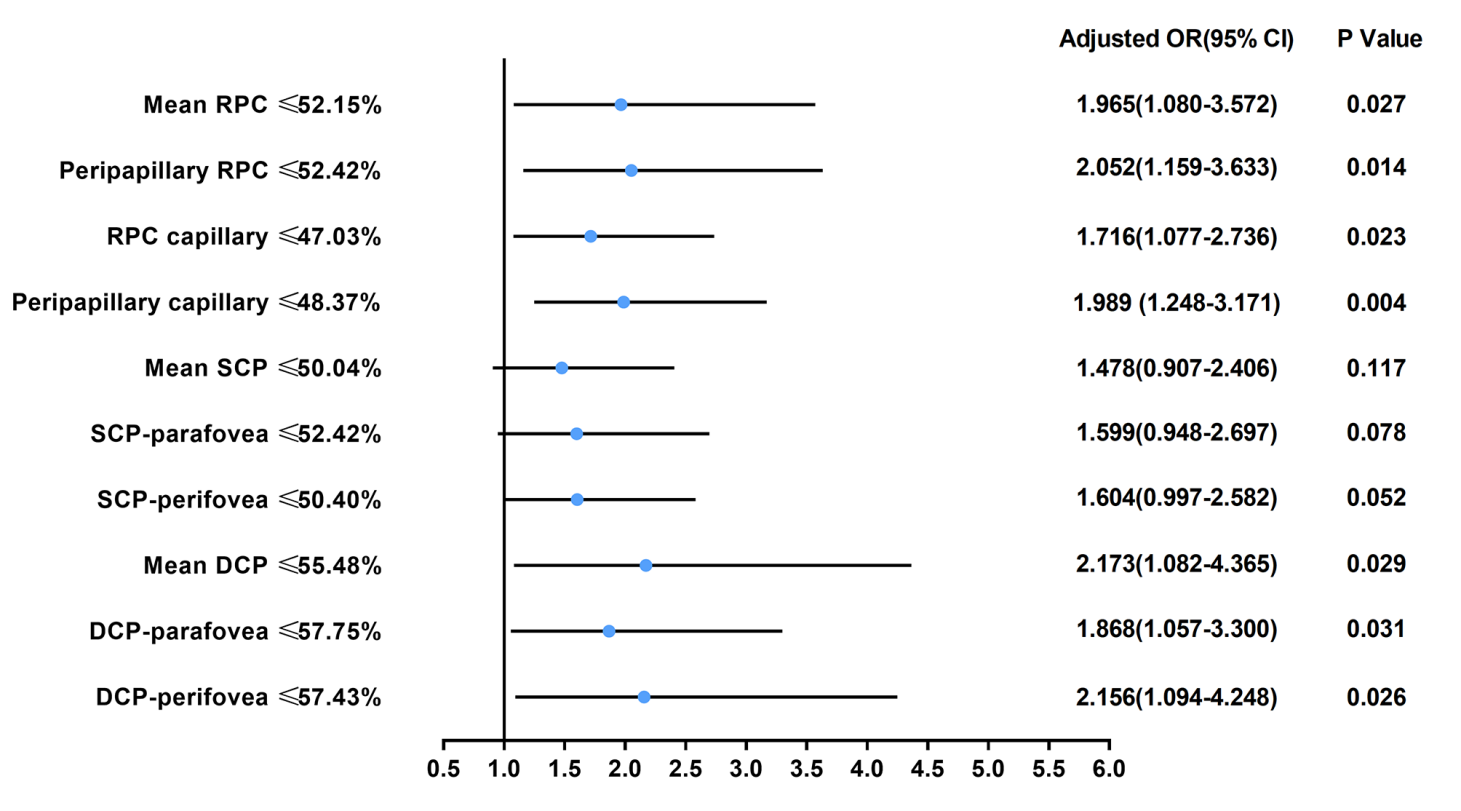
Forest plot for the presence of composite adverse outcomes. Adjusted variables: age, sex, body mass index, cyanosis, previous sternotomy, STAT mortality category and cardiopulmonary bypass time Abbreviations: RPC, radial peripapillary capillary; SCP, superficial capillary plexus; DCP, deep capillary plexus; OR, odds ratio; CI, confidence intervals; STAT, Society of Thoracic Surgeons-European Association for Cardio-Thoracic Surgery Congenital Heart Surgery

The clinical characteristics of patients with CHD with or without composite adverse outcomes are summarized in Supplementary Table S2. Compared with patients without composite adverse outcomes, patients with composite adverse outcomes had lower BMI, a higher proportion of cyanosis and previous sternotomy, longer operation time, CPB time, and aortic cross-clamp time (all *P* < 0.001). Furthermore, patients with composite adverse outcomes had a longer ICU stay, mechanical ventilation time, and postoperative length of stay (all *P* < 0.001). With respect to RVD, the VDs of the RPC, RPC capillary, and SCP were significantly decreased in patients with the composite adverse outcomes (all *P* < 0.05). The VD of the DCP was comparable between patients with and without composite adverse outcomes (all *P* > 0.05) (Table 4). The association between RVD and the composite adverse outcomes is shown in Table 5. After adjusting for confounding factors, lower VD of RPC (OR, 1.97; 95% CI, 1.08–3.57; *P* = 0.027), peripapillary RPC (OR, 2.05; 95% CI, 1.16–3.63; *P* = 0.014), RPC capillary (OR, 1.72; 95% CI, 1.08–2.74; *P* = 0.023), peripapillary capillary (OR, 1.99; 95% CI, 1.25–3.17; *P* = 0.004), DCP (OR, 2.17; 95% CI, 1.08–4.37; *P* = 0.029), DCP-parafovea (OR, 1.87; 95% CI, 1.06–3.30; *P* = 0.031), and DCP-perifovea (OR, 2.16; 95% CI, 1.09–4.25; *P* = 0.026) were significantly associated with the presence of composite adverse outcomes, while no significant association was found between VD of SCP and composite adverse outcomes (Fig. 3).

**Table 4:** Comparison of preoperative RVD in patients with and without composite adverse outcomes

	Patients with composite adverse outcomes (n = 46)	Patients without composite adverse outcomes (n = 127)	**P** value
**RPC density, %**			
Mean	54.05 ± 3.58	55.31 ± 2.92	0.020*
Peripapillary	55.13 ± 4.20	56.64 ± 3.56	0.021*
**RPC capillary density, %**			
Mean	46.18 ± 4.44	48.00 ± 3.34	0.014*
Peripapillary	47.03 ± 5.30	49.17 ± 4.24	0.016*
**Macular vessel density, %**			
Mean SCP	49.71 ± 2.96	50.78 ± 2.55	0.021*
SCP-parafovea	52.30 ± 3.19	53.09 ± 2.98	0.036*
SCP-perifovea	50.39 ± 3.15	51.49 ± 2.79	0.029*
Mean DCP	51.32 ± 5.25	53.10 ± 5.63	0.063
DCP-parafovea	55.27 ± 4.58	56.79 ± 4.83	0.067
DCP-perifovea	52.63 ± 5.78	54.48 ± 5.98	0.071

Abbreviations: RVD, retinal vessel density; RPC, radial peripapillary capillary; SCP, superficial capillary plexus; DCP, deep capillary plexus

*Significant statistical difference, *P* < 0.05

**Table 5:** Association between binary retinal vessel density and the presence of composite adverse outcomes

	Model 1		Model 2		Model 3	
	OR (95% CI)	P value	OR (95% CI)	P value	OR (95% CI)	*P* value
**Mean RPC, %**						
High (> 52.15)	Reference		Reference		Reference	
Low (≤ 52.15)	2.04 (1.24–3.36)	0.005*	1.91 (1.19–3.07)	0.008*	1.97 (1.08–3.57)	0.027*
**Peripapillary RPC, %**						
High (> 52.42)	Reference		Reference		Reference	
Low (≤ 52.42)	2.35 (1.46–3.78)	< 0.001*	2.12 (1.34–3.35)	0.001*	2.05 (1.16–3.63)	0.014*
**RPC capillary, %**						
High (> 47.03)	Reference		Reference		Reference	
Low (≤ 47.03)	2.71 (1.27–3.40)	0.003*	1.89 (1.18–3.03)	0.008*	1.72 (1.08–2.74)	0.023*
**Peripapillary capillary, %**						
High (> 48.37)	Reference		Reference		Reference	
Low (≤ 48.37)	2.23 (1.35–3.67)	0.002*	2.19 (1.33–3.60)	0.002*	1.99 (1.25–3.17)	0.004*
**Mean SCP, %**						
High (> 50.04)	Reference		Reference		Reference	
Low (≤ 50.04)	1.63 (0.99–2.68)	0.053	1.51 (0.95–2.41)	0.084	1.48 (0.91–2.41)	0.117
**SCP-parafovea, %**						
High (> 52.42)	Reference		Reference		Reference	
Low (≤ 52.42)	1.79 (1.03–3.11)	0.038*	1.47 (0.87–2.50)	0.149	1.60 (0.95–2.70)	0.078
**SCP-perifovea, %**						
High (> 50.40)	Reference		Reference		Reference	
Low (≤ 50.40)	1.67 (1.02–2.74)	0.042*	1.59 (1.00-2.52)	0.052	1.60 (1.00-2.58)	0.052
**Mean DCP, %**						
High (> 55.48)	Reference		Reference		Reference	
Low (≤ 55.48)	2.46 (1.23–4.92)	0.011*	2.38 (1.23–4.61)	0.010*	2.17 (1.08–4.37)	0.029*
**DCP-parafovea, %**						
High (> 57.75)	Reference		Reference		Reference	
Low (≤ 57.75)	2.02 (1.13–3.62)	0.018*	2.02 (1.16–3.52)	0.013*	1.87 (1.06–3.30)	0.031*
**DCP-perifovea, %**						
High (> 57.43)	Reference		Reference		Reference	
Low (≤ 57.43)	2.33 (1.17–4.67)	0.017*	2.32 (1.21–4.44)	0.011*	2.16 (1.09–4.25)	0.026*

Model 1: univariate; Model 2: adjusted for age, sex and body mass index; Model 3: adjusted each factor for model 2 plus cyanosis, previous sternotomy, STAT mortality category and cardiopulmonary bypass time

Retinal vessel density is divided into two categories according to cut-off points at the point of maximal sum of sensitivity and specificity using the receiver operating characteristic (ROC) curve

Abbreviations: RPC, radial peripapillary capillary; SCP, superficial capillary plexus; DCP, deep capillary plexus; OR, odds ratio; CI, confidence intervals; STAT, Society of Thoracic Surgeons-European Association for Cardio-Thoracic Surgery

*Significant statistical difference, *P* < 0.05

## Discussion

In this study, we investigated the association between preoperative RVD measured using OCTA and the perioperative outcomes of CHD patients undergoing cardiac surgery with CPB. The major findings of the current study were that lower VD of the DCP was independently associated with the presence of excessive postoperative bleeding, and lower VD of the RPC and DCP were independently associated with the presence of postoperative composite adverse outcomes. This highlights the potential role of OCTA in identifying patients with CHD who are at a high risk of having poor surgical outcomes.

We report several significant findings that could advance our understanding of the value of retinal microcirculation in the assessment of cardiac surgical outcomes. Our results revealed that preoperative retinal hypoperfusion was significantly associated with excessive postoperative bleeding, which is consistent with previous findings that microcirculatory assessment could be a valuable tool to identify patients with a high risk of excessive bleeding after cardiac surgery [5]. There are several possible mechanisms underlying the association between retinal microcirculation and postoperative bleeding. On one hand, hypoperfusion in the DCP might result from the failing circulatory and/or hematological state of CHD patients, which could be related to adverse perioperative outcomes. On the other hand, preoperative microcirculatory disturbances are related to postoperative platelet dysfunction including decreased platelet reactivity as well as elevated platelet adhesion and aggregation in patients undergoing cardiac surgery, resulting in excessive postoperative bleeding [25]. Moreover, microcirculation is the primary site of hemostasis and coagulation, and microvascular endothelial cells can express anti-thrombogenic molecules and pro-coagulants to promote the process of blood coagulation and fibrinolysis [26], suggesting that microcirculatory dysfunction could increase the risk of excessive bleeding after cardiac surgery.

Our further analysis revealed that preoperative decreased RVD was independently associated with postoperative composite adverse outcomes, including operative mortality, early postoperative complications, and prolonged length of stay. In keeping with our findings, microvascular dysfunction is reportedly independently correlated with perioperative complications and poor clinical outcomes in patients undergoing cardiac surgery [6, 7, 27, 28]. Notably, microcirculatory dysfunction could result in tissue hypoperfusion, which is one of the earliest warning signs in critically ill patients, and is considered a predictor of organ ischemia and postoperative adverse clinical outcomes [29, 30]. However, the change in microvascular perfusion was independent of the change in systemic hemodynamics in patients with cardiac surgery [31]. For this reason, it is essential to monitor the microcirculation of patients undergoing cardiac surgery, even when systemic hemodynamics are within satisfactory goals.

The vasculature of the eye shares similar features with the vasculature of the heart and is often exposed to the same intrinsic and environmental influences [32]. The retinal microcirculation can serve as a surrogate measure of the health of the systemic vasculature. An improvement in microcirculation after surgery was reported in CHD patients [16], which further confirmed the retinal microvasculature could reflect the cardiovascular status. Additionally, in this study, we also found an association between retinal microcirculation and perioperative outcomes. The anomaly of the retinal microcirculation might reflect the systemic status of CHD patients, but the exact mechanisms were not clear. More evidence is needed to clarify the association.

Notably, the VD of the DCP rather than the SCP was significantly associated with perioperative outcomes. This phenomenon might be explained by the earlier microvascular damage related to adverse outcomes occurring in DCP than in SCP. Anatomically, the DCP consists of vessels smaller and thinner than the SCP and is mainly enveloped by pericytes, which are more sensitive to ischemia and hypoxia secondary to hypoperfusion [33]. We previously demonstrated that DCP was significantly decreased in cyanotic CHD patients compared to acyanotic CHD patients [15]. Physiologically, the proximity of the DCP to the choriocapillaris may result in more dramatic changes in oxygen tension in the outer retina and correspondingly larger changes in the perfusion of those capillaries [34]. Therefore, hypoperfusion in the DCP was significantly associated with adverse outcomes.

In addition, we found lower VD of the RPC was associated with the presence of postoperative composite adverse outcomes but the association did not appear in the excessive postoperative bleeding. RPC network is a unique vascular plexus in the RNFL and plays a critical role in satisfying the nutritional requirements of retinal ganglion cell (RGC) axons and maintaining neuronal health [35]. Histologically, the RPC network displays minimal intercapillary anastomosis and shows a linear course in keeping with the nerve fiber layer distribution. The anatomic distribution and unique morphologic characteristics help to distinguish the RPC from other capillary plexuses within the retinal microcirculation [36–38]. The retinal vasculature has significant morphologic and functional heterogeneity, which may contribute to the preferential involvement of retinopathy in one retinal region over another in diseases [34]. For example, the DCP is susceptible in the early phases of diabetic retinopathy and hypertension, and RPC plays an important role in the onset and progression of glaucoma. Similarly, in our study, we found RPC was only associated with the presence of composite adverse outcomes, which might imply the RPC could be used as a biomarker to identify the high-risk population of the composite adverse outcomes. The peculiar anatomic conformation and physiological characteristics might be able to explain this phenomenon, but the exact mechanisms for the association between RPC and postoperative outcomes remain unclear. More investigations are needed to clarify the underlying mechanism of the relationship between retinal microcirculation and perioperative outcomes of CHD.

Retinal microvasculature could provide incremental information about the concurrent cardiovascular disease status and predict the future risk of cardiovascular-related events, and retinal imaging could be used as a novel non-invasive screening, diagnostic, and prognostic tool for cardiovascular diseases [39, 40]. One implication of this study is the possible use of a non-invasive and convenient microcirculatory examination, OCTA, to evaluate individual clinical outcomes of CHD patients undergoing cardiac surgery. OCTA can be used to frame therapeutic decisions regarding patient care. For example, patients with retinal hypoperfusion may benefit from more specific hemodynamic management. Additionally, poor perioperative outcomes can place significant financial and psychological pressure on households and the healthcare system. If we can estimate the outcomes related to risk factors before surgery, physicians can tailor interventions for patients to improve their quality of care and reduce the cost of healthcare [41, 42]. Further work in a larger group of CHD patients is required to develop and validate an outcome prediction model integrating preoperative retinal microcirculation.

This study comprehensively investigated the association between retinal microcirculation and perioperative outcomes in patients with CHD undergoing cardiac surgery using a noninvasive and convenient tool. Nonetheless, we acknowledge several limitations of this study. First, CHD patients with STAT category 5 were not included in the study for safety reasons, which could inevitably lead to selection bias. Second, the OCTA technique currently cannot be used in critically ill patients at the bedside. In the future, a portable handheld OCTA instrument can be used to overcome this limitation [43]. Third, the cut-off values for RVD were determined by ROC analyses in our single-center study, which was not representative of other studies. Fourth, we could not take into account all confounding factors during surgery. Furthermore, using composite adverse outcomes in the study may not fully capture individual-specific risks or minor events, warranting careful interpretation of the results and potential further studies to understand the impact of specific adverse event types. Finally, relatively elderly pediatric congenital heart disease patients and a small sample of CHD patients were studied, which might not represent the whole perspective of congenital heart disease and limited power for non-significant associations. Moreover, we selected the CHD patients based on extensive exclusion criteria, which might limit the generalizability of the study findings. Further studies with large-scale sample sizes are needed to corroborate and extend our findings.

## Conclusion

Our study results showed that preoperative retinal hypoperfusion was independently associated with perioperative adverse outcomes in patients with CHD undergoing cardiac surgery, suggesting that retinal microcirculation assessment could provide additional valuable information about the outcomes of cardiac surgery and aid physicians in tailoring individualized treatment plans. Further studies are needed to demonstrate the benefits of retinal microcirculation evaluation in clinical practice.

### Electronic supplementary material

Below is the link to the electronic supplementary material.

Supplementary Material 1

## Data Availability

The original contributions presented in the study are included in the article/Supplementary Material, further inquiries can be directed to the corresponding author/s.
