# Supplementary material for: Association of preoperative retinal microcirculation and perioperative outcomes in patients undergoing congenital cardiac surgery
Source: Orphanet J Rare Dis. 2023 Dec 8;18:385. doi: 10.1186/s13023-023-02969-y (PMC10704768; doi:10.1186/s13023-023-02969-y)
Supplement: Supplementary file 1 — Supplementary Material 1 [file 13023_2023_2969_MOESM1_ESM.docx]

**Supplementary Material**

| **Supplementary Table S1. Comparison of clinical characteristic in patients with and without excessive postoperative bleeding** | | | |
| --- | --- | --- | --- |
|  | **Bleeder (n = 43)** | **Non-bleeder (n = 130)** | **P value** |
| Age, years | 27±11 | 25±13 | 0.426 |
| Gender, n (%) | | | 0.761 |
| Male | 19(44.20) | 54(41.5) |  |
| Female | 24(55.80) | 76(58.5) |  |
| Body mass index, kg/m^2^ | 18.75±3.94 | 18.92±3.60 | 0.792 |
| Previous sternotomy, % | 5(11.6) | 18(13.8) | 0.710 |
| Preoperative SaO_2_, % | 96(86-100) | 99(94-100) | 0.079 |
| Cyanosis, n (%) | 16(37.2) | 28(21.5) | **0.041*** |
| Preoperative laboratory parameters |  |  |  |
| Hb, g/L | 155(132-180) | 140(129-158) | **0.013*** |
| HCT, L/L | 0.47(0.40-0.52) | 0.41(0.38-0.46) | **0.004*** |
| APTT, s | 42.85(39.18-45.20) | 40.80(37.52-43.45) | 0.080 |
| PT, s | 14.70(13.90-15.80) | 14.20(13.60-15.00) | 0.082 |
| TT, s | 16.55(15.90-17.10) | 16.45(15.70-17.18) | 0.879 |
| INR | 1.14(1.06-1.25) | 1.10(1.03-1.16) | **0.047*** |
| Fibrinogen, g/L | 2.45(2.12-3.03) | 2.56(2.25-2.97) | 0.403 |
| STAT mortality category, n (%) | | | 0.770 |
| 1 to 3 | 34(79.1) | 100(76.9) |  |
| 4 | 9(20.9) | 30(23.1) |  |
| Operation time, minutes | 265(206-321) | 225(174-282) | **0.016*** |
| CPB time, minutes | 158(113-192) | 113(82-150) | **0.001*** |
| ACC time, minutes | 93(63-122) | 63(37-90) | **<0.001*** |
| 24-hour CTO, mL | 600(460-875) | 250(155-323) | **<0.001*** |
| ICU stay, days | 3(2-6) | 2(1-3) | **<0.001*** |
| Mechanical ventilation time, hours | 18(7-29) | 6(3-17) | **<0.001*** |
| Postoperative length of stay, days | 9(7-15) | 7(5-11) | **0.002*** |
| Continuous variables are expressed as mean ± standard deviation or median (interquartile range), and categorical variables are expressed as percentages of total.  Abbreviations: SaO_2_, oxygen saturation; Hb, haemoglobin; HCT, haematocrit; APTT, activated partial thromboplastin time; PT, prothrombin time; TT, thrombin time; INR, international normalized ratio; STAT, Society of Thoracic Surgeons-European Association for Cardio-Thoracic Surgery; CPB, cardiopulmonary bypass; ACC, aortic cross-clamp; CTO, chest tube output; ICU, intensive care unit.  *Significant statistical difference, P < 0.05. | | | |

| **Supplementary Table S2. Comparison of clinical characteristic in patients with and without composite adverse outcomes** | | | |
| --- | --- | --- | --- |
|  | **Patients with composite adverse outcomes (n = 46)** | **Patients without composite adverse outcomes**  **(n = 127)** | **P value** |
| Age, years | 26±13 | 25±13 | 0.880 |
| Gender, % | | | 0.623 |
| Male | 18(39.10) | 55(43.3) |  |
| Female | 28(60.90) | 72(56.7) |  |
| Body mass index, kg/m^2^ | 17.07±2.86 | 19.54±3.73 | **<0.001*** |
| Previous sternotomy, n (%) | 13(28.3) | 10(7.9) | **<0.001*** |
| Preoperative SaO_2_, % | 88(81-99) | 99(96-100) | **<0.001*** |
| Cyanosis, % | 24(52.2) | 20(15.7) | **<0.001*** |
| STAT mortality category, n (%) | | | 0.795 |
| 1 to 3 | 35(76.1) | 99(78.0) |  |
| 4 | 11(23.9) | 28(22.0) |  |
| Operation time, minutes | 289(233-350) | 210(169-267) | **<0.001*** |
| CPB time, minutes | 158(123-192) | 111(80-148) | **<0.001*** |
| ACC time, minutes | 90(64-115) | 63(37-91) | **<0.001*** |
| ICU stay, days | 6(2-10) | 2(1-2) | **<0.001*** |
| MV time, hours | 23(9-56) | 6(3-16) | **<0.001*** |
| PLOS, days | 15(12-21) | 6(5-8) | **<0.001*** |
| Continuous variables are expressed as mean ± standard deviation or median (interquartile range), and categorical variables are expressed as percentages of total.  Abbreviations: SaO_2_, oxygen saturation; STAT, Society of Thoracic Surgeons-European Association for Cardio-Thoracic Surgery; CPB, cardiopulmonary bypass; ACC, aortic cross-clamp; CTO, chest tube output; ICU, intensive care unit.  *Significant statistical difference, P < 0.05. | | | |


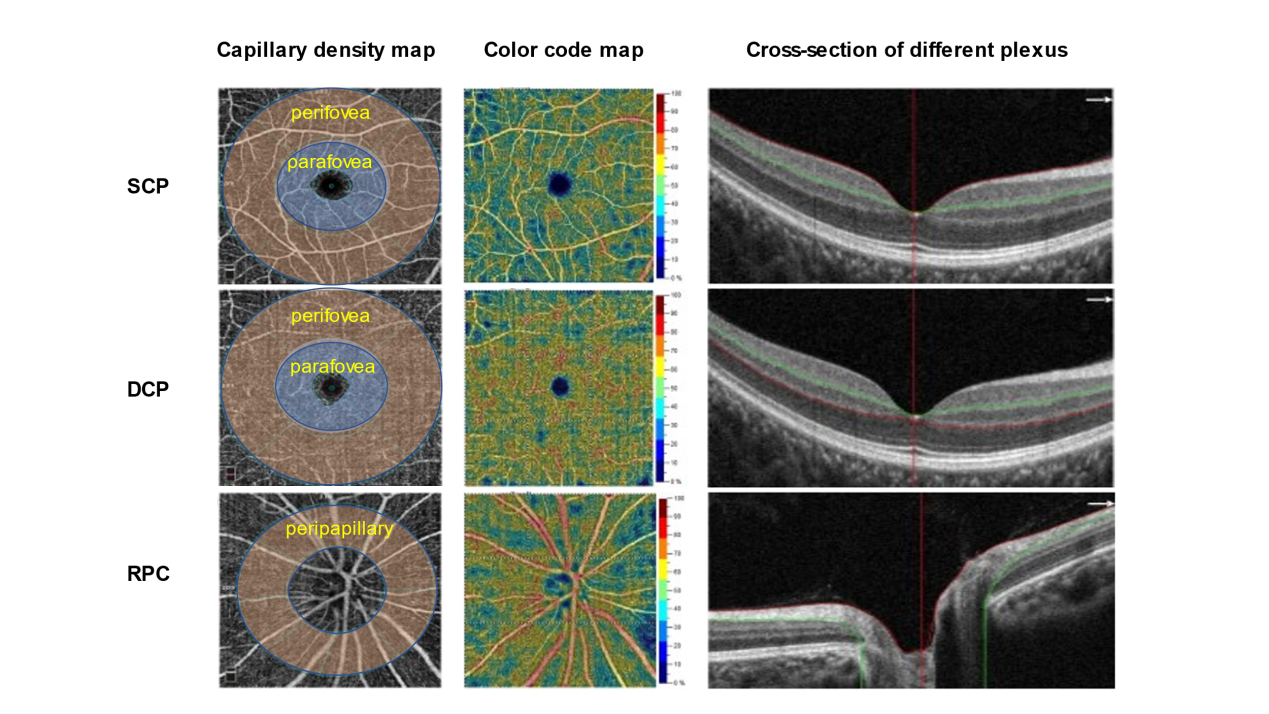


**Supplementary Figure S1.** A representative optical coherence tomography angiography image of different vascular plexus and regions.

The color codes indicates vessel density over the retinal image area. The color scale legend at right associates the colors on the retinal map with percent density in ten percent increments.

Abbreviations: SCP, superficial capillary plexus; DCP, deep capillary plexus; RPC, radial peripapillary capillary.


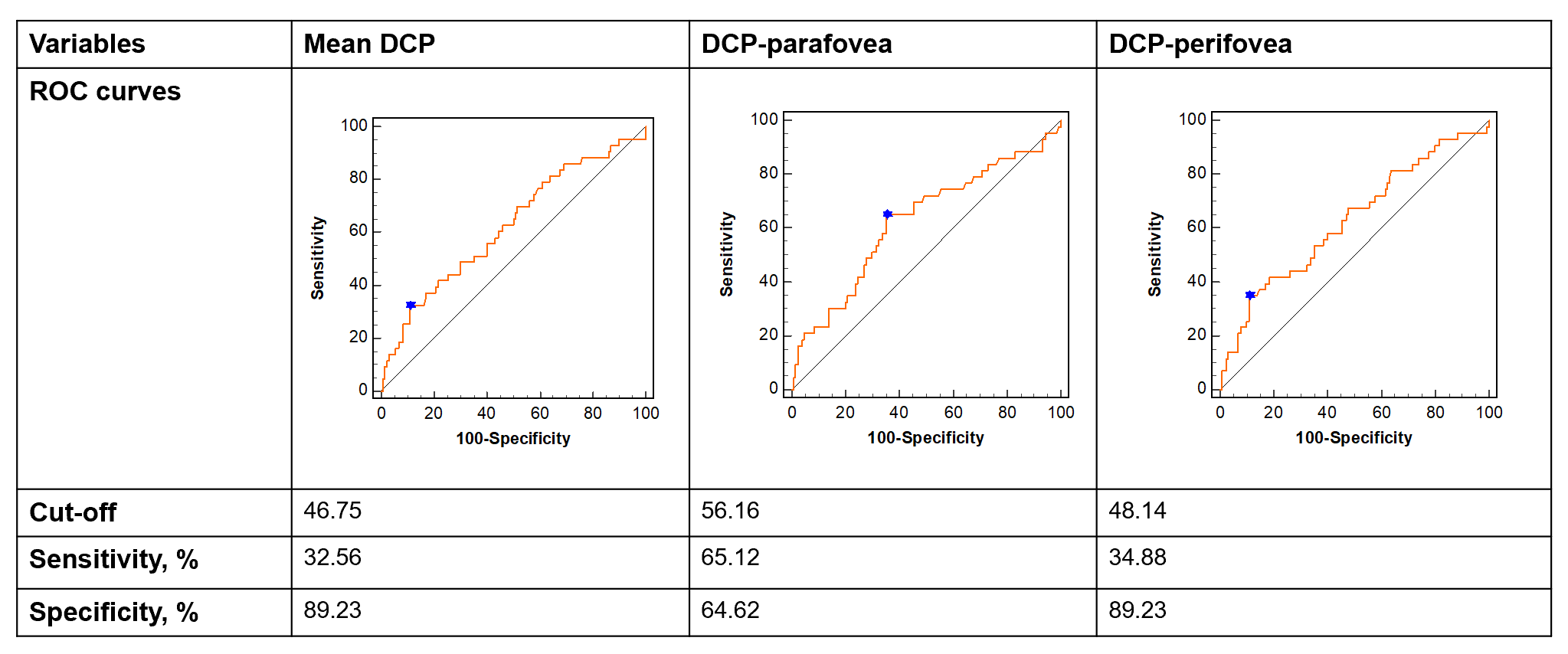


**Supplementary Figure S2.** Dichotomization of RVD based on the ROC curve for excessive postoperative bleeding.

Abbreviations: ROC, receiver operating characteristic; RVD, retinal vessel density; DCP, deep capillary plexus.


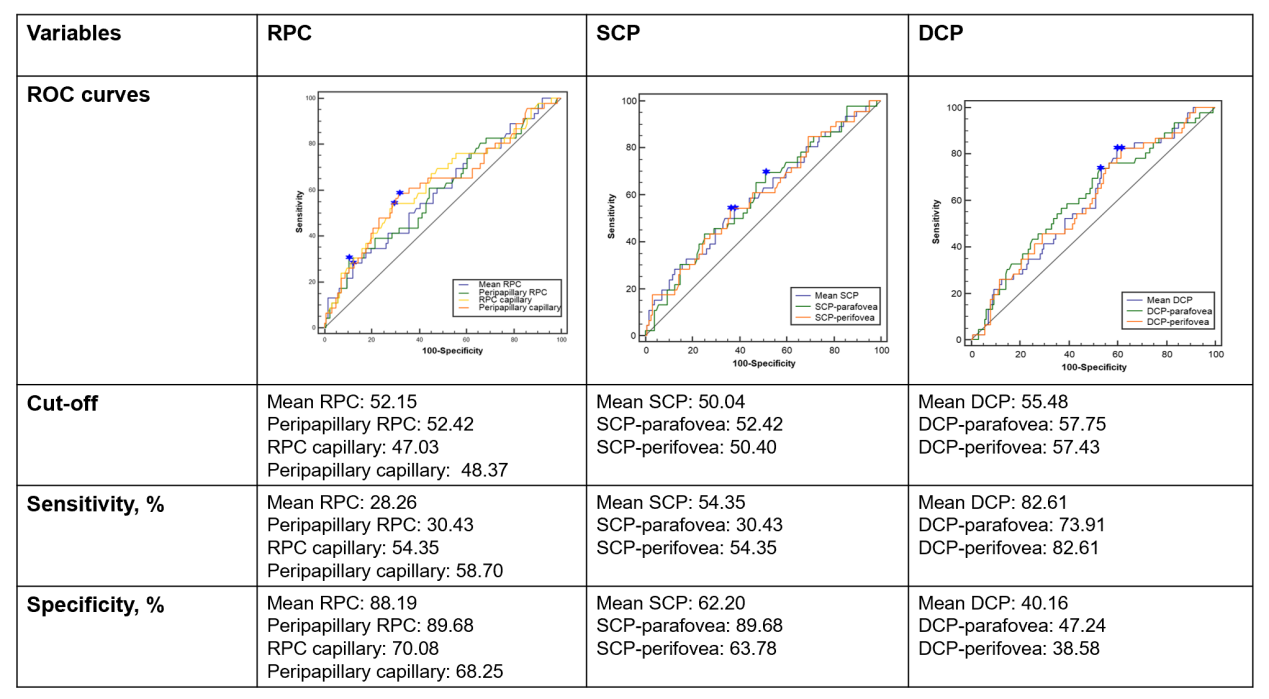


**Supplementary Figure S3.** Dichotomization of RVD based on the ROC curve for composite adverse outcomes.

Abbreviations: ROC, receiver operating characteristic; RVD, retinal vessel density; RPC, radial peripapillary capillary; SCP, superficial capillary plexus; DCP, deep capillary plexus.
